# The Development of an Instrument for Longitudinal Learning Diagnosis of Rational Number Operations Based on Parallel Tests

**DOI:** 10.3389/fpsyg.2020.02246

**Published:** 2020-09-02

**Authors:** Fang Tang, Peida Zhan

**Affiliations:** Department of Psychology, College of Teacher Education, Zhejiang Normal University, Jinhua, China

**Keywords:** learning diagnosis, longitudinal assessments, rational number operations, parallel tests, longitudinal cognitive diagnosis

## Abstract

The precondition of the measurement of longitudinal learning is a high-quality instrument for longitudinal learning diagnosis. This study developed an instrument for longitudinal learning diagnosis of rational number operations. In order to provide a reference for practitioners to develop the instrument for longitudinal learning diagnosis, the development process was presented step by step. The development process contains three main phases, the Q-matrix construction and item development, the preliminary/pilot test for item quality monitoring, and the formal test for test quality control. The results of this study indicate that (a) both the overall quality of the tests and the quality of each item are good enough and that (b) the three tests meet the requirements of parallel tests, which can be used as an instrument for longitudinal learning diagnosis to track students’ learning.

## Introduction

In recent decades, with the development of psychometrics, learning diagnosis (Zhan, 2020b) or cognitive diagnosis (Leighton and Gierl, 2007), which objectively quantifies students’ current learning status, has drawn increasing interest. Learning diagnosis aims to promote students’ learning according to diagnostic results which typically including diagnostic feedback and interventions. However, most existing cross-sectional learning diagnoses are not concerned about measuring growth in learning. By contrast, longitudinal learning diagnosis evaluates students’ knowledge and skills (collectively known as latent attributes) and identifies their strengths and weaknesses over a period (Zhan, 2020b).

A complete longitudinal learning diagnosis should include at least two parts: an instrument for longitudinal learning diagnosis of specific content and a longitudinal learning diagnosis model (LDM). The precondition of the measurement of longitudinal learning is a high-quality instrument for longitudinal learning diagnosis. The data collected from the instrument for longitudinal learning diagnosis can provide researchers with opportunities to develop longitudinal LDMs that can be used to track individual growth over time. Additionally, in recent years, several longitudinal LDMs have been proposed, for review, see Zhan (2020a). Although the usefulness of these longitudinal LDMs in analyzing longitudinal learning diagnosis data has been evaluated through some simulation studies and a few applications, the development process of an instrument for longitudinal learning diagnosis is rarely mentioned (cf. Wang et al., 2020). The lack of an operable development process of instrument hinders the application and promotion of longitudinal learning diagnosis in practice and prevents practitioners from specific fields to apply this approach to track individual growth in specific domains.

Currently, there are many applications use cross-sectional LDMs to diagnose individuals’ learning status in the field of mathematics because the structure of mathematical attributes is relative clear to be identified, such as fraction calculations (Tatsuoka, 1983; Wu, 2019), linear algebraic equations (Birenbaum et al., 1993), and spatial rotations (Chen et al., 2018; Wang et al., 2018). Some studies also apply cross-sectional LDMs to analyze data from large-scale mathematical assessments (e.g., George and Robitzsch, 2018; Park et al., 2018; Zhan et al., 2018; Wu et al., 2020). However, most of these application studies use cross-sectional design and cannot track the individual growth of mathematical ability.

In the field of mathematics, understanding rational numbers is crucial for students’ mathematics achievement (Booth et al., 2014). Rational numbers and their operations are one of the most basic concepts of numbers and mathematical operations, respectively. The fact that many effects are put into rational number teaching makes many students and teachers struggle to understand rational numbers (Cramer et al., 2002; Mazzocco and Devlin, 2008). The content of rational number operation is the first challenge that students encounter in the field of mathematics at the beginning of junior high school. Learning rational number operation is not only the premise of the subsequent learning of mathematics in junior high school but is also an important opportunity to cultivate students’ interest and confidence in mathematics learning.

The main purpose of this study is to develop an instrument for longitudinal learning diagnosis, especially for the content of rational number operations. We present the development process step by step to provide a reference for practitioners to develop the instrument for longitudinal learning diagnosis.

## Development of the Instrument for Longitudinal Learning Diagnosis

**FIGURE 1:**
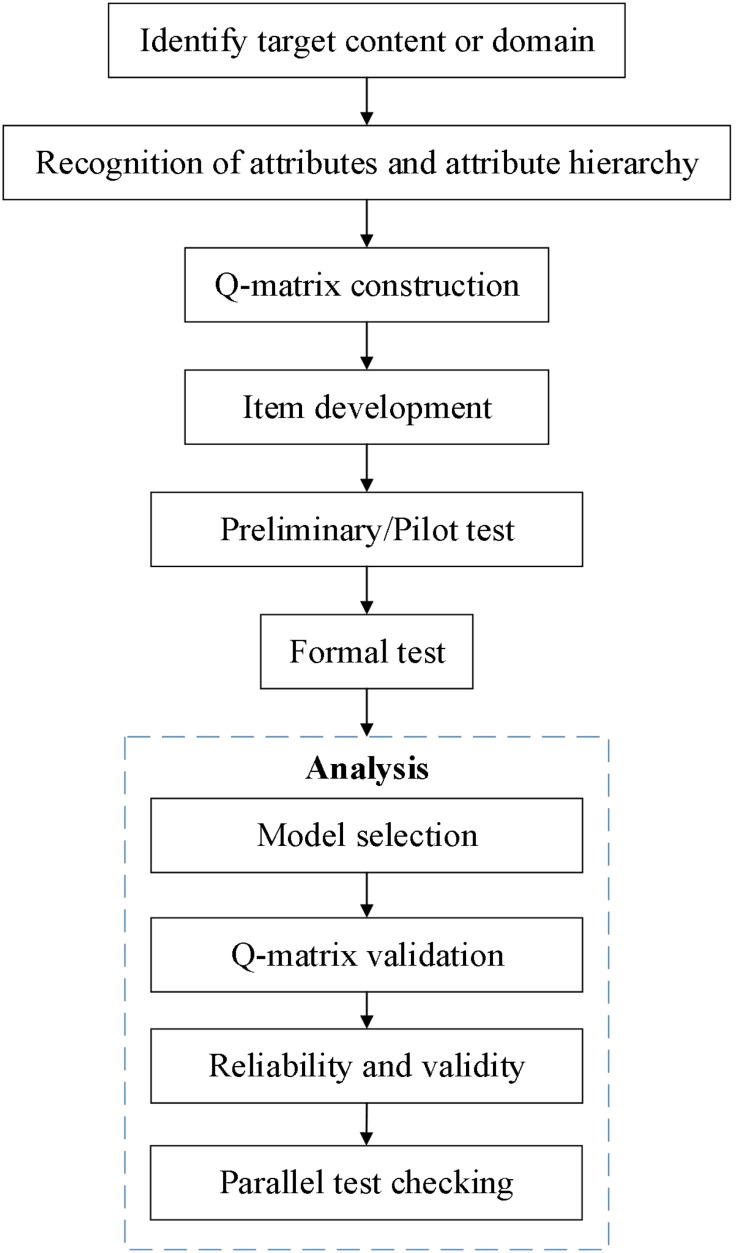
The development process of the instrument for longitudinal learning diagnosis.

As the repeated measures design is not always feasible in longitudinal educational measurement, in this study, the developed instrument is a longitudinal assessment consisting of parallel tests. The whole development process is shown in Figure 1. In the rest of the paper, we describe the development process step by step.

### Recognition of Attributes and Attribute Hierarchy

The first step in designing and developing a diagnostic assessment is recognizing the core attributes involved in the field of study (Bradshaw et al., 2014). In the analysis of previous studies, the confirmation of attributes mainly adopted the method of literature review (Henson and Douglas, 2005) and expert judgment (Buck et al., 1997; Roduta Roberts et al., 2014; Wu, 2019). This study used the combination of these two methods.

First, relevant content knowledge was extracted according to the analysis of mathematics curriculum standards, mathematics exam outlines, teaching materials and supporting books, existing provincial tests, and chapter exercises. By reviewing the literature, we find that the existing researches mainly focus on one or several parts of rational number operation. For example, fraction addition and subtraction is the most involved in existing researches (e.g., Tatsuoka, 1983; Wu, 2019). In contrast, it is not common to focus on the whole part of rational number operation in diagnostic tests. Ning et al. (2012) pointed out that rational number operation contains 15 attributes; however, such a larger number of attributes does not apply in practice.

**FIGURE 2:**
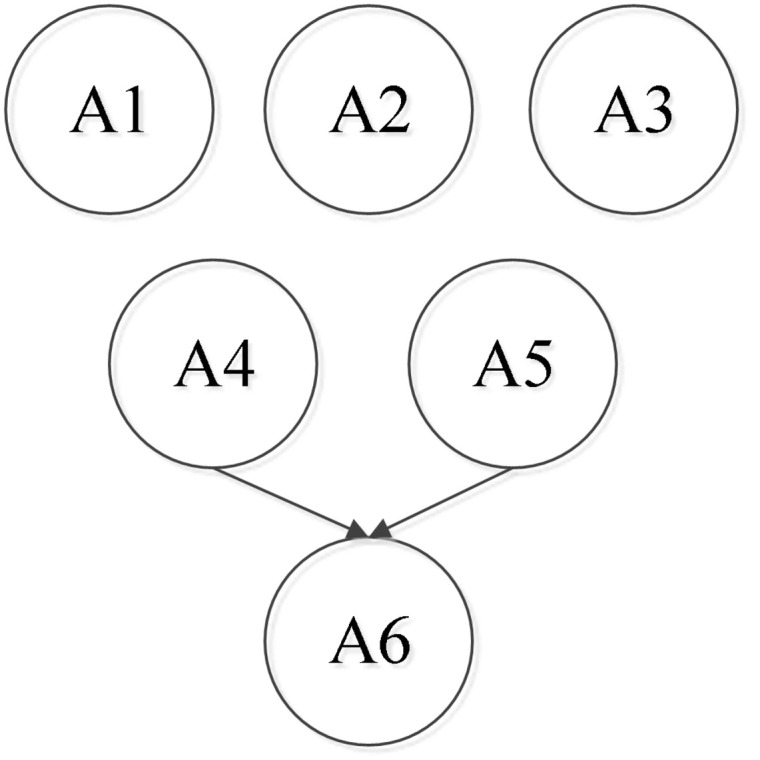
Attribute hierarchy of the rational number operation. Note that A1 = rational number; A2 = related concepts of rational numbers; A3 = axis; A4 = addition and subtraction of rational numbers; A5 = multiplication and division of rational numbers; and A6 = mixed operation of rational numbers.

Second, according to the attribute framework based on the diagnosis of mathematics learning among students in 20 countries in the Third International Math and Science Study–Revised (Tatsuoka et al., 2004), the initial attribute framework and the corresponding attribute hierarchy (Leighton et al., 2004) of this study were determined after a discussion among six experts, including two frontline mathematics teachers who have more than 10 years’ experience in mathematics education, two graduate students majoring in mathematics, and two graduate students majoring in psychometrics (see Table 1 and Figure 2).

**TABLE 1:** Attribute framework of the rational number operation.

Label	Attribute	Description
A1	Rational number	Concepts and classifications
A2	Related concepts of the rational number	Opposite number, absolute value
A3	Number axis	Concept, number conversion, comparison of the size of numbers
A4	Addition and subtraction of rational numbers	Addition, subtraction, and addition operation rules
A5	Multiplication and division of rational numbers	Multiplication, involution, multiplication operation rule, division and reciprocal; Reduction of fractions to a common denominator
A6	Mixed operation of rational numbers	First involution, then multiplication and division, and finally addition and subtraction; if there are numbers in parentheses, calculate the ones in the parentheses first.

**FIGURE 3:**
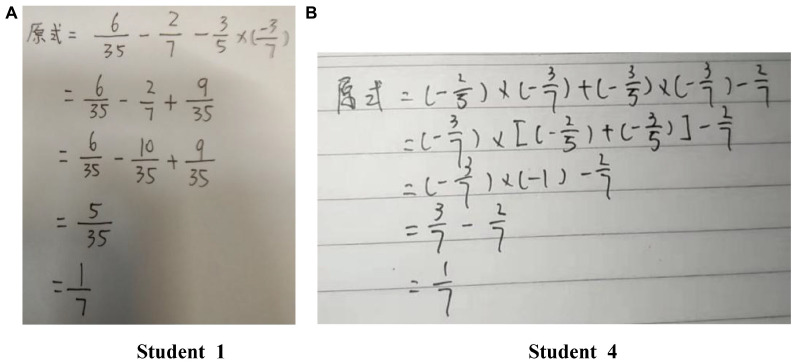
**(A,B)** Problem-solving process of two students in the think-aloud protocol analysis. Note that in item 6, $\left(-\frac{2}{5}\right)\times \left(-\frac{3}{7}\right)-\frac{2}{7}-\frac{3}{5}÷\left(-\frac{7}{3}\right)$with the required attribute pattern (000111).

Third, a reassessment by another group of eight experts (frontline mathematics teachers) and the think-aloud protocol analysis (Roduta Roberts et al., 2014) were used to verify the rationality of the initial attribute framework and that of the corresponding attribute hierarchy. All experts agreed that the attributes and their hierarchical relationships were reasonable. In the think-aloud protocol analysis, six items were initially prepared according to the initial attribute framework and attribute hierarchy (see Table 2). Then, six seventh graders were selected according to above-average performance, gender balance, willingness to participate, and ability to express their thinking process (Gierl et al., 2008). The experimenter individually tested these students and recorded their responses; in the response process, the students were required to say aloud their problem-solving train of thought. Taking the responses of two students to item 6 as an example, Figure 3 and Table 3 present their problem-solving process and thinking process, respectively. Although different students used different problem-solving processes, they all used addition, subtraction, multiplication, and division to solve the items of the mixed operation of rational numbers. Therefore, mastering A4 and A5 are prerequisites to mastering A6, and they validate the rationality of the attribute hierarchy proposed by experts.

**TABLE 2:** Items in think-aloud protocol analysis (original items are written in Chinese).

Please**say out aloud** your thoughts when you solve the problem.
(1) Which one of the following statement about rational numbers is correct? ().
(A) Rational numbers can be divided into two categories: positive rational numbers and negative rational numbers
(B) The set of positive integers and the set of negative integers together constitute the set of integers
(C) Integers and fractions are collectively called rational numbers
(D) Positive numbers, negative numbers, and zeros are collectively called rational numbers
(2) Which rational number’s inverse equals to itself? ().
(A) () 1 (B) −1 (C) 0 (D) 0 and 1
(3) On the number axis, point A indicates −1. Now A starts to move, first move 3 units to the left, then 9 units to the right, and 5 units to the left. At this time, what the number is point A indicates? ().
(A) −1 (B) 0 (C) 1 (D) 8
(4) Computing: 9 + (−13)−(−7) + (−5) =
(5) Computing: $\left(-2\right)\times 14÷\left(-\frac{1}{7}\right)\times {\left(-1\right)}^{5}=$
(6) Computing: $\left(-\frac{2}{5}\right)\times \left(-\frac{3}{7}\right)-\frac{2}{7}-\frac{3}{5}÷\left(-\frac{7}{3}\right)=$

**TABLE 3:** The thinking process of two students in think-aloud protocol analysis.

Student 1:
Step 1: Read the item, and judge that the content knowledge investigated in this item is the mixed operation of rational numbers;
Step 2: Recall the rule for mixed operation of rational numbers: First power, then multiplication and division, final addition and subtraction; If there are parentheses, count them in parentheses first;
Step 3: Make sure multiply and divide first: $\left(-\frac{2}{5}\right)\times \left(-\frac{3}{7}\right)=\frac{6}{35}$, and change division by $\left(-\frac{7}{3}\right)$ to multiply by$\left(-\frac{3}{7}\right)$;
Step 4: Use multiplication: $\left(-\frac{3}{5}\right)\times \left(-\frac{3}{7}\right)=\frac{9}{35}$;
Step 5: Use addition, and get the answer:
**Student 4:**
Step 1: Read the item, and judge that the content knowledge investigated in this item is the mixed operation of rational numbers;
Step 2: Recall the rule for mixed operation of rational numbers: First power, then multiplication and division, final addition and subtraction; If there are parentheses, count them in parentheses first;
Step 3: Observe dividing by $\left(-\frac{7}{3}\right)$ can be changed to multiplying by $\left(-\frac{3}{7}\right)$, the multiplication distribution law can be used;
Step 4: Use the multiplication distribution law, put $\left(-\frac{3}{7}\right)$outside of the parentheses, then $\left(-\frac{2}{5}\right)+\left(-\frac{3}{5}\right)=\left(-1\right)$ in the parentheses;
Step 5: Use subtraction, and get the answer.

*Item 6: $\left(-\frac{2}{5}\right)\times \left(-\frac{3}{7}\right)-\frac{2}{7}-\frac{3}{5}÷\left(-\frac{7}{3}\right)$with required attribute pattern (000111).*

Finally, as presented in Table 1, the attributes of rational number operation fell into the following six categories: (A1) rational number, (A2) related concepts of rational numbers, (A3) axis, (A4) addition and subtraction of rational numbers, (A5) multiplication and division of rational numbers, and (A6) mixed operation of rational numbers. The six attributes followed a hierarchical structure (Figure 2), which indicates that A1–A3 are structurally independent and that A4 and A5 are both needed to master A6.

### Q-Matrix Construction and Item Development

**FIGURE 4:**
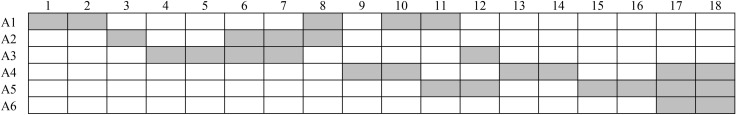
Q-matrix, where blank means “0” and gray means “1.” Note that A1 = rational number; A2 = related concepts of rational numbers; A3 = axis; A4 = addition and subtraction of rational numbers; A5 = multiplication and division of rational numbers; and A6 = mixed operation of rational numbers.

According to the attribute hierarchy, A4 and A5 are both needed to master A6. Therefore, the attribute patterns that contain A6 but lack either A4 or A5 are unattainable. Theoretically, there are 40 rather than 2^6^ = 64 attainable attribute patterns. Correspondingly, the initial Q-matrix (i.e., test blueprint) (Tatsuoka, 1983) was constructed based on these 40 permissible attribute patterns and with the following factors in mind: (a) the Q-matrix contains at least one reachability matrix for completeness (Ding et al., 2010); (b) each attribute is examined at least twice, and (c) the test time is limited to a teaching period of 40 min to ensure that students have a high degree of involvement. Finally, the test length was determined as 18, including 12 multiple-choice items and 6 calculation items (see Figure 4). Notice that all items are dichotomous scored in current study. To ensure that the initial item bank contains enough items, we prepared 4–5 items for each of the 18 attribute patterns contained in the initial Q-matrix. Finally, an initial item bank containing 80 items was formed.

### Preliminary Test: Item Quality Monitoring

#### Participants

In the preliminary test, 296 students (145 males and 151 females) were conveniently sampled from six classes in grade seven of junior high school A^1^.

#### Procedure

To avoid the fatigue effect, 80 items were divided into two tests (preliminary test I and preliminary test II, with 40 items in each test). All participants took part in the two tests. Each test lasted for 90 min, and the two tests were completed within 48 h.

#### Analysis

Item difficulty and discrimination were computed based on the classical test theory. The differential item functioning (DIF) was checked using the difR package (version 5.0) (Magis et al., 2018) in R software.

#### Results

A total of 296 students took the preliminary test. After data cleaning, 270 and 269 valid tests were collected in preliminary test I and preliminary test II, respectively. The effective rates of preliminary test I and preliminary test II were 91.22 and 91.19%, respectively. Table 4 presents the basic sample information and descriptive statistics of the raw scores. The distribution of the raw scores for the two tests was the same.

**TABLE 4:** Basic sample information and descriptive statistics of raw scores in the preliminary test.

Preliminary test	Male	Female	Total	Average score
I	133	137	270	14.77 (8.59)
II	133	136	269	14.78 (8.33)

*Note that each test has a full mark of 40. The standard deviation is indicated in parentheses.*

Table 5 presents the average difficulty and the average discrimination of the preliminary test (the difficulty and discrimination of each item are presented in Table 6). In classical test theory, item difficulty (i.e., the pass rate) is equal to the ratio of the number of people who have a correct response to the total number of people, and item discrimination is equal to the difference between the pass rate of the upper 27% of the group and that of the lower 27% of the group. In general, a high-quality test should have the following characteristics: (a) the average difficulty of the test is 0.5, (b) the difficulty of each item is between 0.2 and 0.8, and (c) the discrimination of each item is greater than 0.3. Based on the above three criteria, we deleted eight items in preliminary test I and seven items in preliminary test II.

**TABLE 5:** Average difficulty and average discrimination of the preliminary tests (based on classical test theory).

Preliminary test	Average difficulty	Average discrimination
I	0.37 (0.15)	0.52 (0.18)
II	0.37 (0.14)	0.51 (0.19)

*Note that the standard deviation is indicated in parentheses.*

**TABLE 6:** Item difficulty and discrimination of preliminary test (based on classical test theory).

Items	Difficulty		Discrimination	
	Preliminary test I	Preliminary test II	Preliminary test I	Preliminary test II
1	0.56	0.52	0.68	0.66
2	0.56	0.18	0.59	0.15
3	0.47	0.54	0.68	0.67
4	0.37	0.50	0.49	0.70
5	0.18	0.43	0.18	0.51
6	0.65	0.22	0.56	0.64
7	0.75	0.64	0.45	0.63
8	0.32	0.36	0.37	0.37
9	0.13	0.52	0.18	0.75
10	0.48	0.30	0.67	0.40
11	0.41	0.41	0.62	0.51
12	0.50	0.49	0.56	0.59
13	0.37	0.33	0.45	0.41
14	0.38	0.31	0.30	0.36
15	0.26	0.28	0.42	0.26
16	0.30	0.42	0.37	0.41
17	0.28	0.20	0.78	0.38
18	0.27	0.38	0.75	0.51
19	0.32	0.27	0.75	0.78
20	0.21	0.12	0.29	0.19
21	0.26	0.26	0.78	0.74
22	0.23	0.32	0.68	0.82
23	0.21	0.33	0.74	0.42
24	0.14	0.15	0.38	0.22
25	0.65	0.22	0.53	0.67
26	0.54	0.24	0.60	0.12
27	0.47	0.20	0.62	0.75
28	0.27	0.65	0.27	0.62
29	0.28	0.54	0.29	0.62
30	0.37	0.31	0.51	0.51
31	0.33	0.64	0.40	0.58
32	0.53	0.47	0.77	0.63
33	0.17	0.21	0.16	0.26
34	0.40	0.38	0.45	0.48
35	0.50	0.33	0.53	0.32
36	0.19	0.52	0.40	0.79
37	0.40	0.39	0.73	0.44
38	0.38	0.50	0.56	0.52
39	0.41	0.29	0.62	0.25
40	0.27	0.41	0.77	0.73

*Items to be deleted including items 5, 9, 20, 24, 28, 29, 33, and 36 in preliminary test I, and items 2, 15, 20, 24, 26, 33, and 39 in preliminary test II.*

Table 7 presents the results of the DIF testing of the preliminary tests. DIF is an important index to evaluate the quality of an item. If an item has a DIF, it will lead to a significant difference in the scores of two observed groups (male and female) in the case of a similar overall ability. In the preliminary tests, the Mantel-Haenszel method (Holland and Thayer, 1986) was used to conduct DIF testing. Male is treated as the reference group, and female is treated as the focal group. The results indicated that items 28 and 36 in preliminary test I had DIF, and no item in preliminary test II had DIF. According to item difficulty and discrimination in the above analysis, these two items were classified as items to be deleted.

**TABLE 7:** Differential item functioning testing of preliminary test.

Items	Preliminary test I			Preliminary test II		
	*p*	deltaMH	Code	*p*	deltaMH	Code
1	0.9012	0.0296	A	0.9446	−0.0276	A
2	0.1318	1.2167	B	0.9412	−0.0313	A
3	0.9508	0.1292	A	0.9317	0	A
4	0.7133	0.3546	A	0.9368	0	A
5	0.9155	−0.1626	A	0.9365	0	A
6	0.4241	0.7871	A	0.9457	0	A
7	0.9055	−0.182	A	0.9448	−0.0289	A
8	0.2583	1.333	B	0.9368	0	A
9	0.6578	0.4168	A	0.9428	0	A
10	0.1922	−0.9753	A	0.9445	0.0841	A
11	0.8356	−0.2203	A	0.9482	0	A
12	0.9223	0.0304	A	0.9455	0	A
13	0.7281	−0.3246	A	0.9383	0	A
14	0.3409	−0.8385	A	0.9416	0	A
15	0.4766	−0.6529	A	0.9483	0	A
16	0.5684	0.8441	A	0.9343	0	A
17	0.8443	0.4158	A	0.9441	0	A
18	0.7296	0.4761	A	0.9131	0	A
19	0.8582	0.2451	A	0.9269	0	A
20	0.9649	0.2428	A	0.9131	0	A
21	0.3897	1.4042	B	0.9272	0	A
22	0.9251	0.5733	A	0.9442	0	A
23	0.7546	0.5935	A	0.9179	0	A
24	0.9732	0.072	A	0.9145	0	A
25	0.5205	−0.4839	A	0.9448	0	A
26	0.9167	0.0326	A	0.9233	0	A
27	0.2342	0.9039	A	0.8897	0	A
28	**0.0425**	−1.5031	C	0.9448	0	A
29	0.8248	−0.251	A	0.9466	0	A
30	0.6341	0.4249	A	0.9442	0	A
31	0.3118	−0.7995	A	0.9445	0	A
32	0.3405	−0.9153	A	0.9429	−0.0299	A
33	0.7845	−0.3828	A	0.9403	0	A
34	0.4017	0.6477	A	0.9466	−0.0264	A
35	0.2172	−0.9052	A	0.9457	0	A
36	**0.0365**	1.9793	C	0.937	0	A
37	0.3919	−0.746	A	0.9449	0	A
38	0.8351	−0.2381	A	0.9454	−0.0271	A
39	0.8637	−0.2109	A	0.9464	0	A
40	0.1209	1.8533	C	0.9386	0.1058	A

*DIF items in bold (*p* < 0.05); A, B, and C are the codes of effect size (i.e., the absolute value of deltaMH), where A means negligible effect, B means moderate effect, and C means large effect.*

By analyzing item difficulty, item discrimination, and DIF, 65 items finally remained (including 32 items in preliminary test I and 33 items in preliminary test II) to form the final item bank. Among them, there are 3–5 candidate items corresponding to each of the 18 attribute patterns in the initial Q-matrix. Furthermore, based on the initial Q-matrix, three learning diagnostic tests with the same Q-matrix were randomly extracted from the final item bank to form the instrument of the formal tests: formal test A, formal test B, and formal test C.

### Formal Test: Q-Matrix Validation, Reliability and Validity, and Parallel Test Checking

It was possible that the initial Q-matrix was not adequately representative despite the level of care exercised. Thus, empirical validation of the initial Q-matrix was still needed to improve the accuracy of subsequent analysis (de la Torre, 2008). Although item quality was controlled in the preliminary test, it was necessary to ensure that these three tests, as instruments for longitudinal learning diagnosis, met the requirements of parallel tests. Only in this way could the performance of students at different time points be compared.

#### Participants

In the formal tests, 301 students (146 males and 155 females) were conveniently sampled from six classes in grade seven of junior high school B.

#### Procedure

All participants were tested simultaneously. The three tests (i.e., formal tests A, B, and C) were tested in turn. Each test lasted 40 min, and the three tests were completed within 48 h.

#### Analysis

Except for some descriptive statistics, the data in the formal test were mainly analyzed based on the LDMs using the CDM package (version 7.4-19) (Robitzsch et al., 2019) in R software. Including the model–data fitting, the empirical validation of the initial Q-matrix, the model parameter estimation, and the testing of reliability and validity were conducted. In the parallel test checking, the consistency of the three tests among the raw scores, the estimated item parameters, and the diagnostic classifications were calculated.

The deterministic-input, noisy “and” (DINA) model (Junker and Sijtsma, 2001), the deterministic-input, noisy “or” (DINO) model (Templin and Henson, 2006), and the general DINA (GDINA) model (de la Torre, 2011) were used to fit the data. In the model–data fitting, as suggested by Chen et al. (2013), the AIC and BIC were used for the relative fit evaluation, and the RMSEA, SRMSR, MADcor, and MADQ3 were used for the absolute fit evaluation. In the model parameter estimation, only the estimates of the best-fitting model were presented. In the empirical validation of the initial Q-matrix, the procedure suggested by de la Torre (2008) was used. In the model-based DIF checking, the Wald test (Hou et al., 2014) was used. In the testing of reliability and validity, the classification accuracy (*P*_*a*_) and consistency (*P*_*c*_) indices (Wang et al., 2015) were computed.

#### Results

##### Descriptive statistics of raw scores

A total of 301 students took the formal test. After data cleaning, the same 277 valid tests (including those from 135 males and 142 females) were collected from each of the three tests; the effective rate of the formal tests was 93.57%. Table 8 presents the descriptive statistics of raw scores in the formal tests. The average, standard deviation, mode, median, minimum, and maximum of raw scores of the three tests were the same.

**TABLE 8:** Descriptive statistics of raw scores in the formal tests.

Formal test	Average score	Mode	Median	Minimum	Maximum
A	7.24 (4.95)	2	6	0	18
B	7.36 (5.03)	3	6	0	18
C	7.31 (4.98)	2 and 3	6	0	18

*Note that the standard deviation is indicated in parentheses. The tests have a full mark of 18.*

##### Model–data fitting

The parameters in an LDM can be interpreted only when the selected model fits the data. The fit indices presented in Table 9 provide information about the data fit of three LDMs, namely DINA, DINO, and GDINA, to determine the best-fitting model. Absolute fit indices hold that values near zero indicate an absolute fit (Oliveri and von Davier, 2011; Ravand, 2016). The result indices indicated that all three models fitted the data well. For relative fit indices, smaller values indicate a better fit. The DINA model was preferred based on the BIC, and the GDINA model was preferred based on the AIC. According to the parsimony principle (Beck, 1943), a simpler model is preferred if its performance is not significantly worse than that of a more complex model. Both AIC and BIC introduced a penalty for model complexity. However, as the sample size was included in the penalty in BIC, the penalty in BIC was larger than that in AIC. The DINA model was chosen as the best-fitting model given the small sample size of this study, which might not meet the needs of an accurate parameter estimation of the GDINA model, and the item parameters in the DINA model having more straightforward interpretations. Therefore, the DINA model was used for the follow-up model-based analyses.

**TABLE 9:** Relative and absolute model–data fit indices.

Formal test	Model	AIC	BIC	RMSEA	SRMSR	MADcor	MADQ3
A	DINA	4646.96	4918.76	0.038	0.057	0.041	0.055
	DINO	4822.67	5094.47	0.050	0.096	0.066	0.071
	GDINA	4635.77	4994.55	0.057	0.046	0.032	0.057
B	DINA	4843.47	5115.27	0.065	0.061	0.046	0.064
	DINO	4994.34	5266.14	0.048	0.094	0.069	0.067
	GDINA	4834.38	5193.15	0.064	0.054	0.039	0.063
C	DINA	4877.31	5149.11	0.041	0.070	0.048	0.062
	DINO	4975.42	5247.22	0.040	0.093	0.065	0.066
	GDINA	4822.55	5182.24	0.060	0.049	0.049	0.063

##### Q-matrix validation

A misspecified Q-matrix can seriously affect the parameter estimation and the results of diagnostic accuracy (de la Torre, 2008; Ma and de la Torre, 2019). Notice that the Q-matrix validation can also be skipped when the model fits the data well. Table 10 presents the revision suggestion based on the empirical validation of the initial Q-matrix. In all three tests, the revision suggestion was only for item 9. However, after the subjective and empirical judgment of the experts (Ravand, 2016), this revision suggestion was not recommended to be adopted. Let us take item 9 (“Which number minus 7 is equal to −10?”) in formal test A as an example. Clearly, this item does not address the suggested changes in A3, A5, and A6. As the expert-defined Q-matrix was consistent with the data-driven Q-matrix, the initial Q-matrix was used as the confirmed Q-matrix in the follow-up analyses.

**TABLE 10:** Revision suggestion based on the empirical validation of the initial Q-matrix.

Formal test	Item	Initial required attribute pattern						Revision suggestion					
		A1	A2	A3	A4	A5	A6	A1	A2	A3	A4	A5	A6
A	9	0	0	0	1	0	0	0	0	1	1	1	1
B	9	0	0	0	1	0	0	0	1	1	1	1	0
C	9	0	0	0	1	0	0	0	0	1	1	0	0

##### Reliability and validity

Classification accuracy (*P*_*a*_) and consistency (*P*_*c*_) are two important indicators for evaluating the reliability and validity of classification results. According to Ravand and Robitzsch (2018), values of at least 0.8 for the *P*_*a*_ index and 0.7 for the *P*_*c*_ index can be considered acceptable classification rates. As shown in Table 11, both pattern- and attribute-level classification accuracy and consistency were within the acceptable range. Additionally, Cronbach’s α, split-half reliability, and parallel form reliability were also computed based on the raw scores (see Table 12). The attribute framework of this study was reassessed by several experts, and the Q-matrix was confirmed, indicating that the content validity and the structural validity of this study were good. To further verify the external validity, the correlation between the raw score of each formal test and the raw score of a monthly exam (denoted as S; the content of this test is the chapter on “rational numbers”) was computed (*r*_A__S_ = 0.95, *p* < 0.01; *r*_B__S_ = 0.95, *p* < 0.01; *r*_CS_ = 0.94, *p* < 0.01). The results indicated that the reliability and validity of all three tests were good.

**TABLE 11:** Classification accuracy and consistency indices based on the DINA model.

Attributes	Formal test A		Formal test B		Formal test C	
	*P*_*a*_	*P*_*c*_	*P*_*a*_	*P*_*c*_	*P*_*a*_	*P*_*c*_
A1	0.96	0.93	0.95	0.92	0.94	0.90
A2	0.93	0.88	0.98	0.97	0.97	0.94
A3	0.94	0.89	0.96	0.93	0.93	0.87
A4	1.00	1.00	1.00	1.00	1.00	1.00
A5	1.00	1.00	0.99	0.98	0.99	0.99
A6	1.00	1.00	1.00	0.99	1.00	0.99
Attribute pattern	0.85	0.74	0.89	0.84	0.85	0.77

**TABLE 12:** Reliability of formal tests.

Formal test	Cronbach’ α	Split-half reliability
A	0.887	0.907
B	0.889	0.923
C	0.886	0.915
*r*_AB_	0.97**	
*r*_AC_	0.96**	
*r*_BC_	0.96**	

*Split-half reliability is calculated according to the items of odd and even numbers; *r*_*AB*_ = the parallel-forms reliability of tests A and B; *r*_*AC*_ = the parallel-forms reliability of tests A and C; *r*_*BC*_ = the parallel-forms reliability of tests B and C; ^∗∗^*p* < 0.01.*

##### Parallel test checking

To determine whether there were significant differences in the performance of the same group of students in the three tests, the raw scores, estimated item parameters (Table 13), and diagnostic classifications (Table 14) were analyzed by repeated measures ANOVA. The results indicated no significant difference in the raw scores [*F*(2,552) = 1.054, *p* = 0.349, BF_10_ = 0.038^2^], estimated guessing parameters [*F*(2,34) = 1.686, *p* = 0.200, BF_10_ = 0.463], estimated slip parameters [*F*(2,34) = 0.247, *p* = 0.783, BF_10_ = 0.164], and diagnostic classifications [*F*(2,78) ≈ 0.000, *p* ≈ 1.000, BF_10_ = 0.078] in the same group of students in the three tests.

**TABLE 13:** Item parameter estimates in formal tests.

Items	Formal test A		Formal test B		Formal test C	
	*g*	*s*	*g*	*s*	*g*	*s*
1	0.4927	0.0861	0.3972	0.0433	0.3849	0.0348
2	0.0155	0.1101	0.3044	0.1604	0.3588	0.1948
3	0.1046	0.0978	0.0009	0.0412	0.0771	0.0775
4	0.0796	0.0770	0.1431	0.0510	0.1248	0.1016
5	0.1721	0.3809	0.1590	0.1940	0.1702	0.3689
6	0.2260	0.4177	0.2739	0.3422	0.2373	0.4373
7	0.2774	0.1514	0.2868	0.0431	0.2667	0.0669
8	0.1785	0.2215	0.2924	0.2209	0.2915	0.3311
9	0.2827	0.1860	0.2984	0.1746	0.3089	0.1629
10	0.2676	0.3429	0.2605	0.3315	0.2747	0.2124
11	0.2921	0.3247	0.3427	0.3018	0.2739	0.2673
12	0.1314	0.3891	0.2270	0.2827	0.2387	0.2271
13	0.0001	0.0001	0.0001	0.0119	0.0001	0.0468
14	0.0001	0.0233	0.0001	0.0468	0.0001	0.0119
15	0.0443	0.0001	0.0224	0.0417	0.0310	0.0001
16	0.0201	0.0001	0.0201	0.1881	0.0256	0.1310
17	0.0371	0.0711	0.0510	0.2085	0.0464	0.1626
18	0.0093	0.0532	0.0099	0.0403	0.0140	0.0583
Mean	0.1462	0.1630	0.1717	0.1513	0.1736	0.1607

*g = guessing parameter; s = slip parameter.*

**TABLE 14:** Diagnostic classifications of students in formal test.

Attribute pattern	Formal test A		Formal test B		Formal test C	
	Proportion	Number of students	Proportion	Number of students	Proportion	Number of students
000000	20.06%	56	29.13%	81	19.02%	53
100000	0.00%	0	0.00%	0	6.89%	19
010000	19.38%	54	15.00%	42	6.82%	19
110000	6.59%	18	4.64%	13	11.43%	32
001000	6.16%	17	2.61%	7	5.35%	15
101000	0.00%	0	0.43%	1	1.50%	4
011000	0.92%	3	0.86%	2	1.81%	5
111000	12.99%	36	12.72%	35	14.01%	39
000100	1.53%	4	0.92%	3	0.00%	0
100100	0.00%	0	0.47%	1	2.09%	6
010100	0.75%	2	1.59%	4	1.80%	5
110100	2.58%	7	1.63%	5	1.50%	4
001100	0.00%	0	0.19%	1	0.30%	1
101100	1.09%	3	0.87%	2	0.84%	2
111100	0.50%	1	0.49%	1	1.05%	3
100010	0.23%	1	0.29%	1	0.00%	0
010010	0.49%	1	0.77%	2	0.42%	1
110010	0.56%	2	1.16%	3	0.71%	2
011010	0.00%	0	0.00%	0	0.26%	1
111010	1.56%	4	1.34%	4	0.72%	2
100110	2.06%	6	1.85%	5	1.09%	3
010110	0.00%	0	0.21%	1	0.32%	1
110110	0.51%	1	0.54%	1	0.00%	0
101110	0.00%	0	0.32%	1	0.00%	0
111110	0.98%	3	0.62%	2	0.71%	2
101111	0.48%	1	1.55%	4	0.00%	0
111111	20.56%	57	19.83%	55	21.34%	59

*Attribute patterns with 0 person in all three tests are omitted.*

As the three tests examined the same content knowledge, contained the same Q-matrix, had high parallel-forms reliability, and had no significant differences in the raw scores, estimated item parameters, and diagnostic classifications, they could be considered to meet the requirements of parallel tests.

## Conclusion and Discussion

This study developed an instrument for longitudinal learning diagnosis of rational number operations. In order to provide a reference for practitioners to develop the instrument for longitudinal learning diagnosis, the development process was presented step by step. The development process contains three main phases, the Q-matrix construction and item development, the preliminary test for item quality monitoring, and the formal test for test quality control. The results of this study indicate that (a) both the overall quality of the tests and the quality of each item are good enough and that (b) the three tests meet the requirements of parallel tests, which can be used as an instrument for longitudinal learning diagnosis to track students’ learning.

However, there are still some limitations of this study. First, to increase operability, only the binary attributes were adopted. As the binary attribute can only divide students into two categories (i.e., mastery and non-mastery), it may not meet the need for a multiple levels division of practical teaching objectives (Bloom et al., 1956). Polytomous attributes and the corresponding LDMs (Karelitz, 2008; Zhan et al., 2020) can be adopted in future studies. Second, the adopted instrument for longitudinal learning diagnosis was based on parallel tests. However, in practice, perfect parallel tests do not exist. In further studies, the anchor-item design (e.g., Zhan et al., 2019) can be adopted to develop an instrument for longitudinal learning diagnosis. Third, an appropriate Q-matrix is one of the key factors in learning diagnosis (de la Torre, 2008). However, the Q-matrix used in the instrument may not strictly meet the requirements of identification (Gu and Xu, 2019), which may affect the diagnostic classification accuracy.

## Data Availability Statement

The raw data supporting the conclusions of this article will be made available by the authors, without undue reservation.

## Ethics Statement

Ethical review and approval was not required for the study on human participants in accordance with the local legislation and institutional requirements. Written informed consent from the participants’ legal guardian/next of kin was not required to participate in this study in accordance with the national legislation and the institutional requirements.

## Author Contributions

FT conducted data acquisition and analysis. PZ provided the idea, wrote the first draft, and revised the manuscript. Both authors contributed to the article and approved the submitted version.

## Conflict of Interest

The authors declare that the research was conducted in the absence of any commercial or financial relationships that could be construed as a potential conflict of interest.

## Footnotes

1 Three schools were used in the complete study. In the instrument development, students in schools A and B participated in the preliminary test and the formal test, respectively; students in school C participate in the quasi-experiment.

2 The Bayes factor (BF_10_) was calculated using the JASP software (Goss-Sampson, 2020) based on the Bayesian estimation. BF_10_ = 0.038 means that the current data are 0.038 times more likely to occur under the alternative hypothesis (H1) being true than under the null hypothesis (H0) being true. As suggested by Dienes (2014), BF_10_ less than 1, 1/3, and 1/10 represents weak, moderate, and strong evidence for the H0, respectively. By contrast, BF_10_ greater than 1, 3, and 10 represents weak, moderate, and strong evidence for the H1, respectively.
